# Assessing Numerical Dependence in Gene Expression Summaries with the Jackknife Expression Difference

**DOI:** 10.1371/journal.pone.0039570

**Published:** 2012-08-02

**Authors:** John R. Stevens, Gabriel Nicholas

**Affiliations:** 1 Department of Mathematics and Statistics, Center for Integrated Biosystems, Utah State University, Logan, Utah, United States of America; 2 Department of Biostatistics and Medical Informatics, University of Wisconsin, Madison, Wisconsin, United States of America; National Taiwan University, Taiwan

## Abstract

Statistical methods to test for differential expression traditionally assume that each gene's expression summaries are independent across arrays. When certain preprocessing methods are used to obtain those summaries, this assumption is not necessarily true. In general, the erroneous assumption of dependence results in a loss of statistical power. We introduce a diagnostic measure of numerical dependence for gene expression summaries from any preprocessing method and discuss the relative performance of several common preprocessing methods with respect to this measure. Some common preprocessing methods introduce non-trivial levels of numerical dependence. The issue of (between-array) dependence has received little if any attention in the literature, and researchers working with gene expression data should not take such properties for granted, or they risk unnecessarily losing statistical power.

## Introduction

### Background

The expression values of thousands of genes can be monitored simultaneously using microarray technology [1], [2]. Applications of this technology abound in the literature. This paper assumes that the reader is somewhat familiar with this technology, particularly the GeneChip microarray from Affymetrix (www.affymetrix.com), which is the most commonly used platform for gene expression studies. Some common terminology is defined herein only for the sake of clarity.

Preprocessing refers to the steps taken to convert the raw probe-level intensities to a collection of estimates of each gene's expression values on each array [3], [4]. With the Affymetrix platform, preprocessing typically includes background correction (to remove local noise and other small artifacts), normalization (to make inter-array comparisons meaningful), and summarization (to combine probe-level data to a gene-level summary). A variety of preprocessing methods have been proposed for Affymetrix data, with MAS5 [5], [6], Li-Wong (also referred to as dChip, or MBEI for model-based expression index) [7]–[9], RMA [3], [10], GCRMA [11], PLIER [12], [13], and PUMA [14]–[16] among the most commonly used. Each of these methods has a convenient implementation among the Bioconductor tools [17] for the R computing environment [18]. The result of each of these methods can be thought of as a matrix of gene expression estimates (or gene expression summaries), with a row for each gene and a column for each array in an experiment. Rather than fully summarizing each of these preprocessing methods here, we refer interested readers to the references.

After preprocessing, a wide variety of analysis options are available. When the arrays can be classified by some categorical variable, such as disease state (healthy vs. beginning disease vs. advanced disease, for example) or treatment state (control vs. treatment, for example), a test of differential expression can be considered. A test of significance is conducted to identify individual genes (or groups of genes) that exhibit systematic shifts in expression values between levels of the categorical variable.

While there are perhaps less than a dozen major preprocessing methods in the literature (plus their variants), the number of proposed methods for evaluating differential expression continues to grow. We do not attempt to catalog every possible test here, nor do we claim to have a best test. Instead, we focus our attention on a common assumption in these tests, that a gene's expression summaries from multiple arrays are independent. This is different from the issue of dependence among genes, which has been addressed previously by others [19], [20]. The linear models framework in the limma approach [21] assumes the independence of a gene's expression levels, with any dependence “assumed to be such that it can be ignored to a first order approximation.” Other t-statistic-based approaches such as SAM [22] also implicitly assume this independence.

Depending on the preprocessing method, the expression summaries for a given gene may not be truly independent across arrays. For example, RMA essentially shares information across arrays at both the (quantile) normalization and (median polish) summarization steps, so the RMA expression summaries on one array will depend to some degree on the original intensities on other arrays. On the other hand, MAS5 preprocesses each array individually, sharing no information across arrays at any step of preprocessing.

A general principle of statistical inference is that if model assumptions are violated, no claim of statistical significance can be made. A common goal of statistical applications to gene expression data is to perform statistical inference by identifying significantly differentially expressed genes. We seek to draw attention to the fact that any such statistical inference is suspect when the assumption of independence is violated. Our motivation in this paper is primarily to shed light on the numerical properties of several common gene expression summaries, as they relate to this assumption of independence, rather than to account for dependence in a particular test for differential expression.

### Illustrative Scenario

To illustrate the impact of erroneously assuming independence, we present a small illustrative scenario. We emphasize that this small scenario is merely used to illustrate the principle that ignoring dependence matters in statistical inference, and hope that this scenario does not detract from the main focus of this paper, which is given in the Methods section.

Consider a two-sample 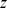-test, where for replicate 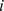 of treatment 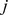 (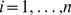; 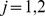),


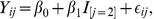
(1)

where the indicator function 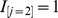 when 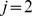, and equals 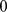 otherwise. Here, the vector 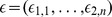 is multivariate normal with mean 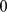 and compound symmetric covariance matrix 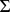:


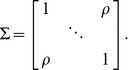
(2)

That is, 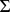 is 1 on the diagonal and 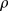 for all off-diagonal elements, with 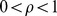 defining the degree of dependence. This scenario can be represented in matrix form:


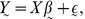
(3)

where 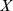 is the 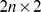 design matrix with all 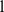
′s in the first column, and with 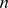 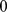
′s followed by 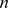 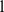
′s in the second column, and 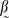 is the vector of “intercept” and “slope” (or “treatment effect”) parameters


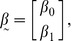
(4)

and 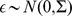 so that 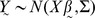, where the vector 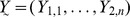.

Using ordinary least squares (i.e., ignoring the dependence 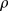) and linear models theory [23],


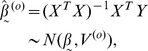
(5)

where 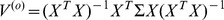 is a 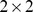 matrix. Here, the 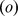 in superscript is for ordinary least squares. It can be shown (using a symbolic computation package such as Maple) that the variance of 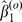 is 
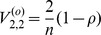
. Based on this ordinary least squares approach (which assumes 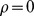), 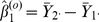, and the 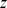-statistic to test 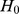: 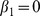 is


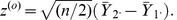
(6)

If 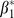 is the true value of 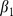 and 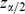 is the upper 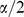 critical value of the standard normal distribution, then the statistical power for the test of 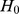: 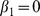 (while ignoring dependence) is


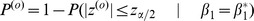



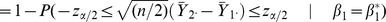



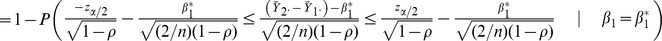



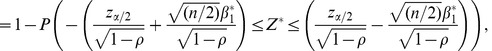
(7)

where 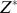 is a truly 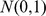 random variable. Specifically, 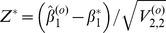.

Using weighted least squares (i.e., accounting for the dependence 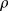) and linear models theory [23],


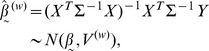
(8)

where 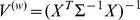 is a 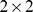 matrix. Here, the 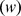 in superscript is for weighted least squares. It can be shown (using a symbolic computation package such as Maple) that the variance of 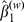 is 
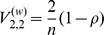
. Based on this weighted least squares approach, 
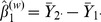
, and the 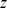-statistic to test 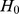: 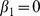 is


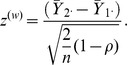
(9)

The statistical power for the test of 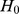: 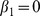 (while accounting for dependence) is


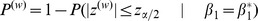



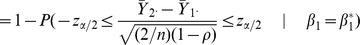



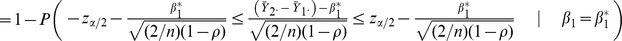



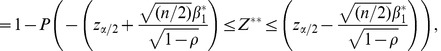
(10)

where 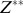 is a truly 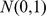 random variable. Specifically, 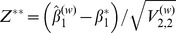.

We can compare the statistical power when dependence is ignored (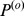 in Equation 7) with the statistical power when dependence is accounted for (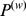 in Equation 10) by focusing on the left and right endpoints of their respective final probability formulae. If 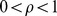, then 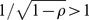, so


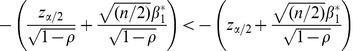
(11)

and


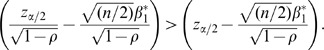
(12)

It follows then that 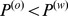.

**Figure 1:**
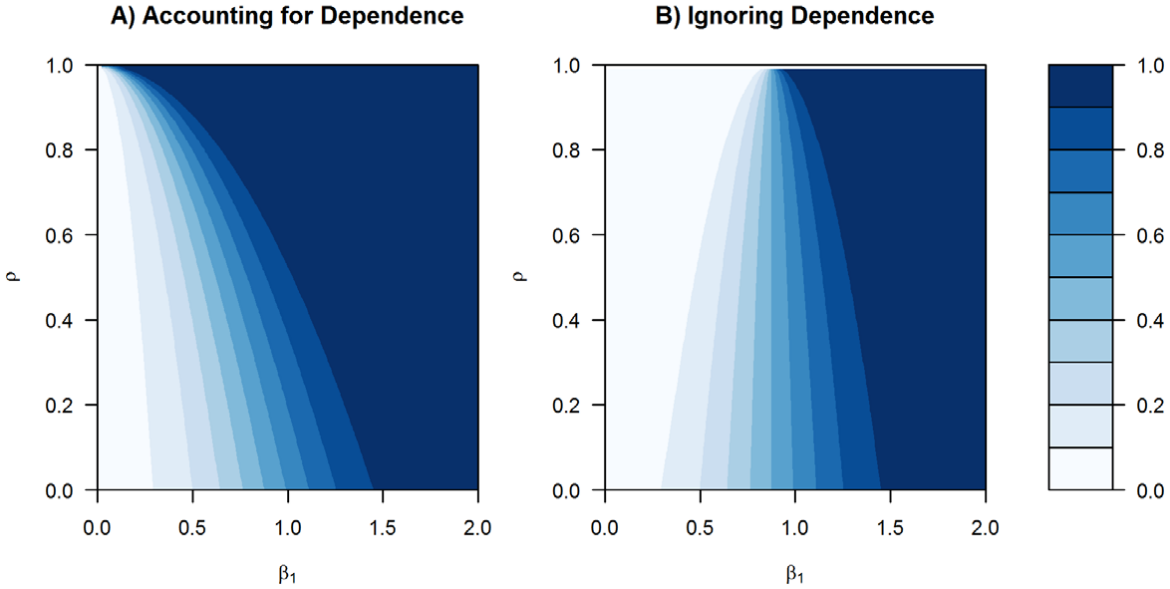
Power Contours From Illustrative Scenario. Ignoring dependence leads to a loss of statistical power, with greater losses for greater dependence (larger 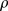) and more subtle magnitudes of differential expression or “treatment effect” (smaller 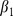). The color scale for statistical power is summarized in the legend at right.

The contour plots in Figure 1 summarize this difference in power for a range of 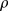 and 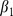 values. Clearly, greater magnitude of “treatment effect” 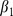 leads to greater statistical power at any given level of dependence 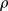. However, ignoring dependence leads to a loss of statistical power, with greater losses for greater dependence (higher 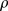) and more subtle magnitudes of “treatment effect” (smaller 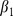). Although the tests for differential expression with real gene expression data may be different than a simple two-sample 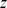-test, this general principle remains – that erroneously assuming independence leads to a loss of statistical power. This motivates our attention to the numerical dependence introduced by various common preprocessing methods.

## Methods

### Jackknife Expression Difference (JED)

Here we propose a simple method to assess the (between-array) numerical dependence of gene expression summaries. For a particular gene, let 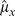 and 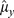 be the gene's non-negative log-scale expression level summaries for arrays 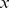 and 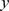, respectively, after some preprocessing method. (For certain preprocessing methods, including PLIER and PUMA, it is possible to find negative expression summaries. We treat such cases as having very little evidence of expression, and reset negative expression summaries on an array to the smallest positive expression summary observed for all genes on the array.) For the same gene, let 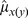 be the expression level estimate for array 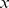 when array 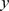 is not included in any step of the preprocessing, with convention 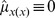 to represent no information for array 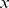 when excluded. Then we define the Jackknife Expression Difference (JED) between arrays 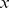 and 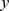 for the gene to be


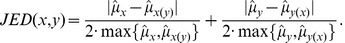
(13)

Notice that by definition, 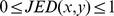, and 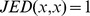, indicating strict numerical dependence of an array (or its summaries) with itself. Also, this JED measure is standardized such that 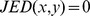 when 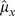 and 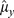 are strictly numerically independent, i.e., when 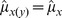 and 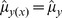. 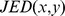 can be interpreted as the average percent change in the expression value of the gene because of the inclusion or exclusion of arrays 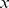 and 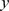 in the preprocessing.

JED values of 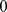 indicate total independence between pairs of arrays, while values of 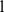 indicate total dependence between pairs of arrays. If 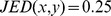 for a particular gene and arrays 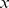 and 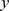, then the expression value of the gene on those two arrays would change by an average 25% if either array had not been included in the study.

Incidentally,


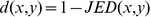
(14)

defines a distance function for the gene's expression summaries between arrays 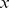 and 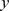. The jackknife approach can be considered the simplest of resampling techniques [24], and while it can exclude more than one at a time, the most common application of the jackknife principle is “leave-one-out” [25]. In the multi-array gene expression situation, this allows for pairwise (between array) distance comparisons by dropping (one at a time) members of pairs of arrays (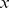 and 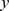). Other resampling approaches such as the general jackknife (leaving out more than one) or the bootstrap (drawing at random with replacement) do not lend themselves so easily to this pairwise interpretation.

The R code to obtain this JED measure is provided (with an example) in Text S1.

### Covariance and JED

The JED measure assesses numerical dependence in gene expression summaries. While similar in spirit, this numerical dependence is not the same as what we refer to as statistical dependence, which could be represented by a true correlation or covariance matrix for each gene. If 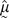 is the vector of expression summaries (for all arrays) for a given gene under a particular preprocessing method, then the covariance matrix would be


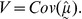
(15)

Constructing such a per-gene covariance matrix for a given preprocessing method would require a well-defined distribution for the method's gene expression summaries 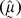. In practice, such well-defined distributions are rare (and unheard of) for preprocessing methods, and it is usually not possible to estimate this matrix 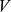. For some preprocessing methods, however, the diagonal elements of 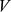 (the variances of the expression summaries) can be estimated, either in closed form based on the distribution of 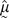 (as for Li-Wong and for PUMA), or as an approximation using the bootstrap (as for RMA [26]).

To investigate the general relationship between a gene's Jackknife Expression Difference 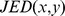 and the covariance 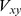 for a pair of arrays 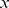 and 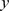, we define a preprocessing method we will refer to as MINDEP (for minimum dependency). We emphasize that we do not recommend using this preprocessing method in general; we only use it here because its resulting covariance matrix 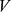 can be obtained using standard statistical theory. In this MINDEP approach, no background correction and no normalization is done, and a two-factor linear ANOVA-type model is assumed for each gene at the summarization step:


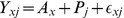
(16)

Here, 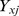 is the log-scale perfect match intensity for probe 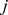 on array 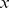, 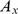 is the mean array effect, and 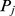 is the mean probe effect. Let 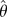 be the vector of resulting ordinary least squares parameter estimates of the 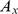
's and 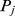
's, with covariance matrix 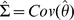. This covariance matrix can be obtained because of the well-known properties of least squares estimates [23]. The LSMEAN (or marginal mean or population mean) for the gene on array 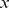 is defined as


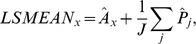
(17)

where 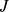 is the number of probes for the gene. Then the MINDEP expression summary for the gene on array 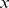 is defined as


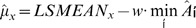



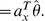
(18)

Here, 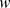 is a weight parameter ranging from 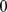 to 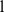, and 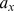 is a vector of appropriate coefficients (specific to array 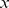).

For the sake of completeness, we briefly show the construction of 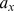 from Equation 18. Let 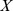 be the number of arrays and 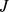 be the number of probes for a given gene. Corresponding to array 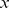, 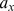 is a length 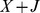 vector with 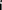
th element 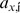. For 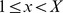, 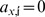 for 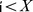 and 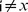, 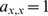, and 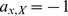. For 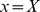, 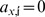 for 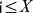. For 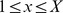 and 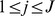, 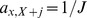. For example, if there were 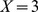 arrays and 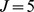 probes, then the three vectors 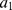, 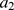, 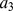 would be the rows of the matrix


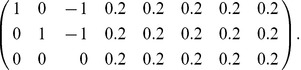
(19)

The subtraction of the minimum array mean in Equation 18 is intended to serve as a pseudo-background-correction, and larger 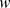 introduces greater dependence between the resulting expression summaries, with known covariance between arrays 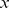 and 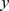:


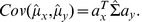
(20)

Thus for each gene, we can obtain a vector of MINDEP expression estimates 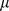 and its corresponding covariance matrix 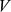. The weight parameter 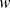 can be varied to show the simultaneous effect of greater dependence on covariance and JED.

We note that the weight parameter 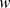 in Equation 18 could be set to give negative covariance values between arrays 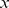 and 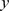. However, by definition (and via the built-in symmetry), JED is non-negative. This helps preserve its interpretation.

## Results

For illustration purposes, we applied this JED measure for six common preprocessing methods to four datasets. The publicly-available Affymetrix HGU95A spike-in data [27] consist of 59 arrays and 12,626 probesets on each array. For our demonstration, only 8 arrays were used, corresponding to groups M-T of wafer 1532 of the spike-in data. We also applied JED to the publicly-available Platinum Spike [28] data set (18,952 probesets on each of 18 arrays) and the publicly-available Golden Spike [29] data set (14,010 probesets on each of 6 arrays) as well as a previously published Asbestos [30] data set (54,675 probesets on each of 6 arrays). Because the results from these four different data sets were so similar, we do not fully report the results from each. Unless otherwise specified, the results given here are for the HGU95A spike-in data.

### Visual Summaries of JED

**Figure 2:**
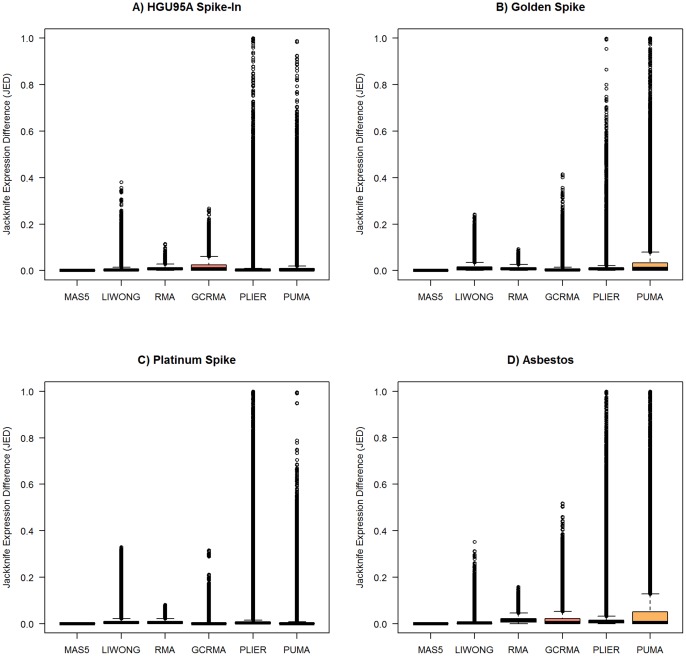
JED: Jackknife Expression Differences for the (A) HGU95A, (B) Golden Spike, (C) Platinum Spike, and (D) Asbestos data. The JED measures for all genes and all pairs of arrays in the four data sets are visualized for each of six common preprocessing methods. 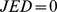 corresponds to numerical independence. For purposes of visualization, 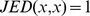 values are suppressed.

Figure 2 summarizes the results for all probesets and all array pairs for the four data sets. For all preprocessing methods considered, 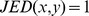 if and only if 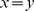, so for purposes of visualization, points corresponding to the same array pair (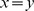, where 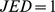) are omitted. The PLIER and PUMA methods produced the most extreme JED measures, while only the MAS5 method demonstrated true numerical independence (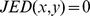 for all 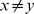). The popular RMA method introduces some numerical dependence, but the dependence is certainly not as substantial as that observed in other methods.

**Figure 3:**
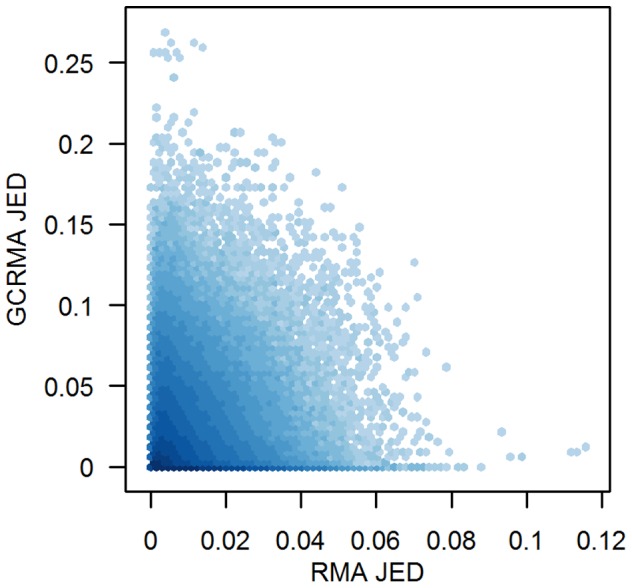
Comparison of JED from RMA and GCRMA. The JED measures for all genes and all pairs of arrays in the example (HGU95A) data set are compared for two preprocessing methods. Darker colors indicate greater density of points. For purposes of visualization, 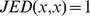 values are suppressed.

**Figure 4:**
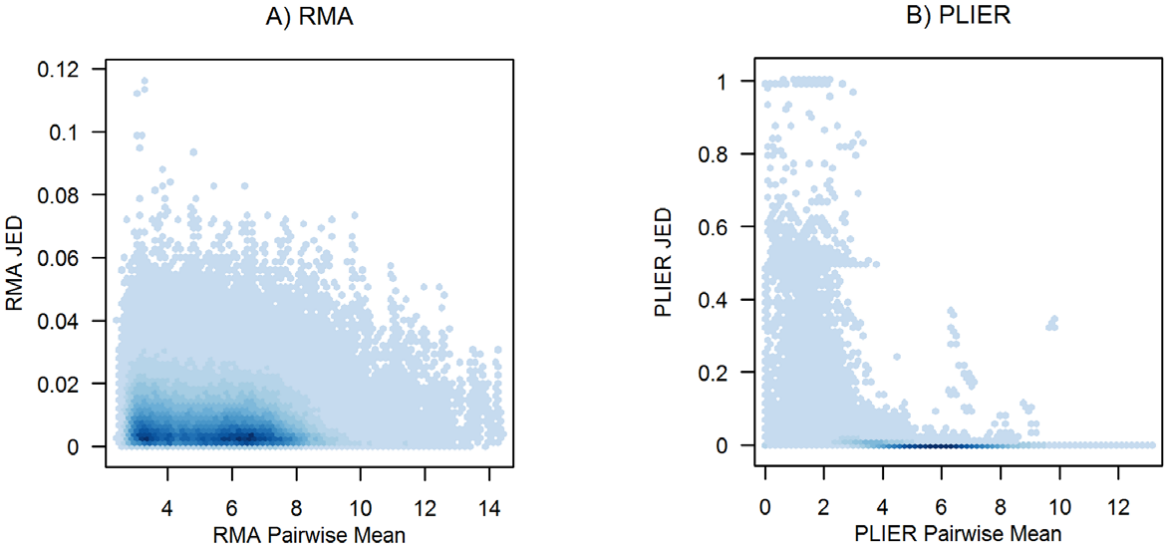
JED and Expression Magnitude for RMA and PLIER. The JED measures for all genes and all pairs of arrays in the example (HGU95A) data set are plotted against the genes' expression summaries, averaged over the corresponding pairs of arrays. For purposes of visualization, 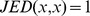 values are suppressed.

We considered whether the JED measure preserves some biological or chemical aspect of the genes. If it did, we would expect to see similarities in JED measures from different preprocessing methods, especially similar preprocessing methods. Figure 3 compares the JED measures for RMA and GCRMA, which share the same quantile approach at the normalization step and the same median polish approach [31] at the summarization step of preprocessing. (Figures 3 and 4 make use of hexagonal binning [32] in the scatter plots, with darker colors indicating greater density of points.) Based on Figure 3, there is no evidence that the JED measures from these two preprocessing methods are related, even though they share two preprocessing steps. Similar non-relation results (not shown) are observed for the other pairs of preprocessing methods that do not share preprocessing steps. This suggests that the JED measure reports numerical artifacts of the preprocessing method, and is not biological or chemical in origin.

We also considered if the magnitude of the JED measure might be related to the corresponding magnitude of expression. Figure 4A compares the JED measure for RMA with the pairwise mean RMA expression summary. That is, for each gene, and for each pair of arrays 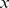 and 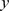, 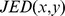 is plotted against 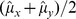. For purposes of visualization, points corresponding to the same array pair (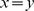, where 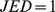) are omitted. The largest JED values correspond to lower-expressed genes, but relatively large JED values can be observed for higher-expressed genes. Similar results are observed for other preprocessing methods, including PLIER as in Figure 4B. We note with some concern that some large PLIER expression values (around 10) have moderately large JED values (around 0.35), such that some of the most highly expressed genes (after PLIER preprocessing) are subject to about 35% average change in expression based on the inclusion or exclusion of some arrays. While the results of Figure 4 are for this sample HGU95A data set, the trends seen here raise concern about the levels of numerical dependence introduced by some preprocessing methods, even for more highly-expressed genes.

### Numerical Artifact Due to Sign Changes

**Figure 5:**
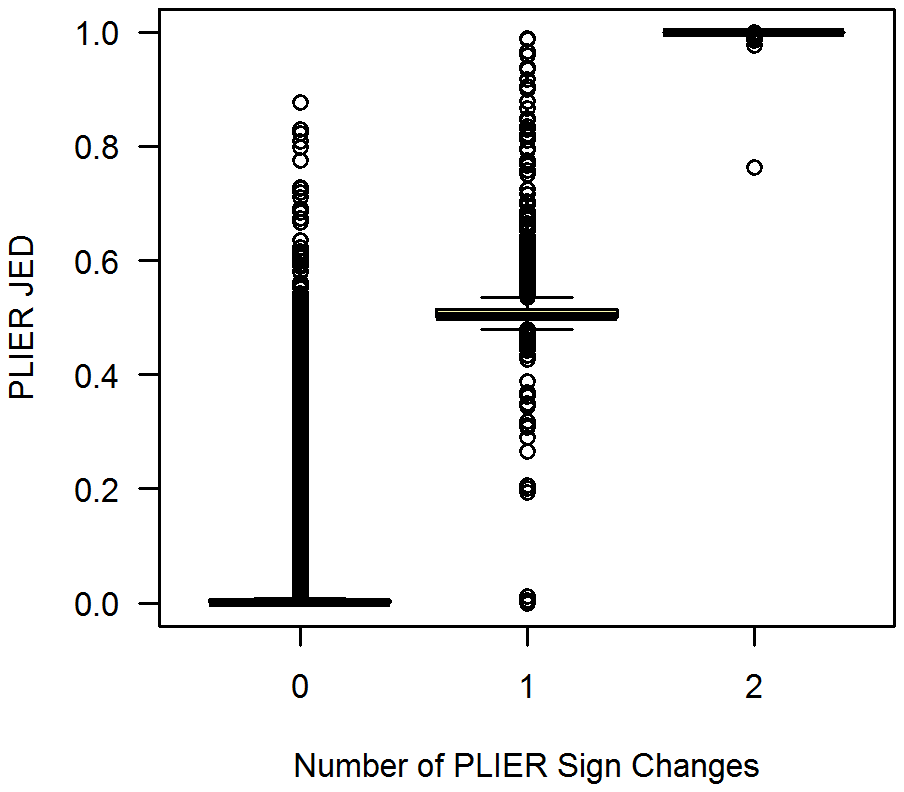
JED and Jackknife Sign Changes for PLIER. The JED measures for all genes and all pairs of arrays in the example (HGU95A) data set are summarized according to the number of sign changes (0, 1, or 2) observed in the expression summaries of the jackknife estimates. For purposes of visualization, 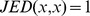 values are suppressed.


Figure 4B shows some banding near PLIER JED values of 0.5 and 1, which are an artifact of sign changes induced by the jackknife. For example, for a given gene and arrays 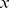 and 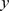, it could be that 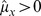 but 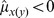, so that the jackknife (exclusion of array 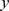) induces a sign change for the gene's expression summary on array 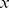. Similarly, exclusion of array 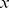 could induce a sign change for the gene's expression summary on array 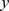. In both cases, the sign change could go from positive to negative or from negative to positive. For each gene and each pair of arrays (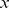, 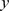), the number of sign changes induced by the jackknife will be 0, 1, or 2. Figure 5 summarizes the PLIER JED values by number of observed sign changes, with clear banding at 0.5 for genes (and array pairs) with one sign change, and at 1 for those with two sign changes. From the Methods section above, recall that we treat a negative expression summary as having very little evidence of expression, and reset such negative expression summaries on an array to the smallest positive expression summary observed for all genes on the array. Let 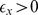 be the smallest positive expression summary observed for all genes on array 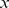, and 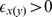 be the smallest positive expression summary observed for all genes on array 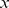 when array 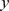 is excluded from the preprocessing. Then if a gene exhibits a sign change on array 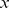 upon exclusion of array 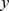 (for example, 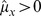, but 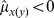 is reset to 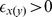), the first portion of the JED calculation in Equation 13 is

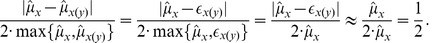
(21)

This explains the pattern near 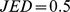 observed for genes (and array pairs) with one sign change in Figure 5. If the gene (and array pair) has two sign changes induced by the jackknife, then both portions of the JED calculation in Equation 13 will be approximately 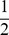 (as in Equation 21), explaining the pattern near 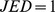 for genes (and array pairs) with two sign changes in Figure 5. It is important to point out that even if one focuses only on genes with positive expression summaries (zero sign changes in Figure 5), very high JED values can be seen for PLIER. Similar results (not shown here) can be seen for PUMA, the other preprocessing method considered here with possibly negative expression summaries.

### JED and Correlation

**Figure 6:**
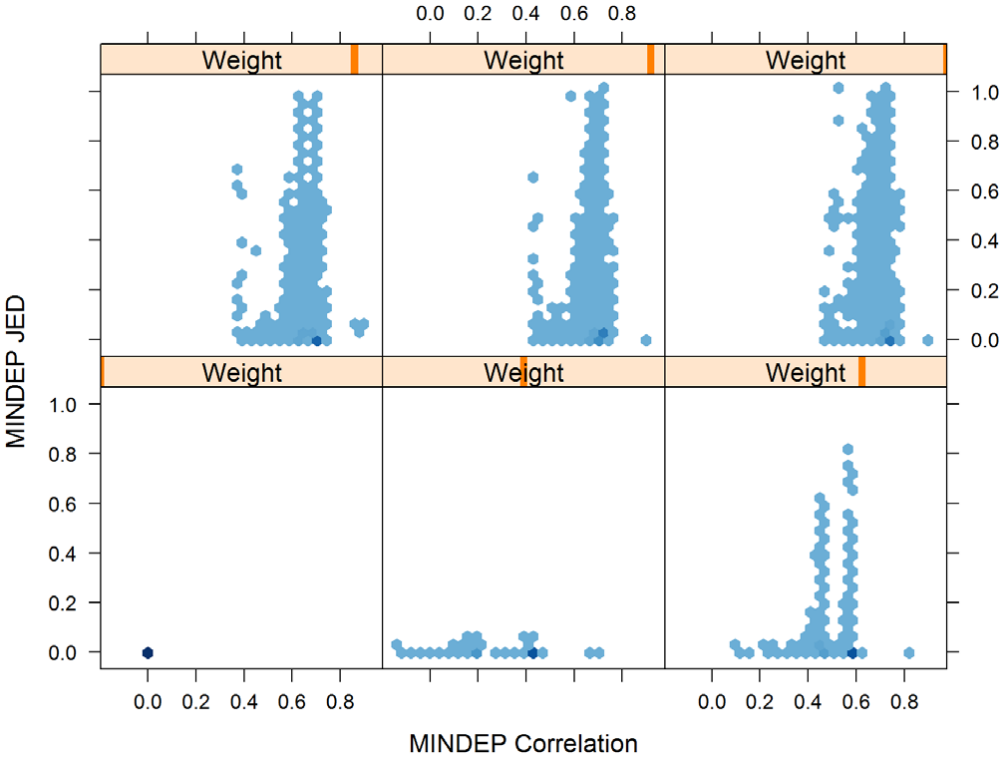
MINDEP JED vs. Correlation by Weight. In this trellis plot, the JED measures for all genes and all pairs of arrays in the example (HGU95A) data set are compared to the corresponding correlations based on the MINDEP preprocessing method, using weight parameter 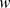 values 0, 0.5, 0.7, 0.9, 0.95, and 1. The value of the weight parameter 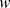 is represented by the position of the colored bar in the “Weight” title (from 0 for far left position to 1 for far right position). Darker colors indicate greater density of points. For purposes of visualization, 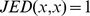 values are suppressed.

Using the previously defined MINDEP preprocessing method, we considered the general relationship between JED and correlation (rescaled covariance) between expression summaries. The trellis plot in Figure 6 summarizes the result. (Like Figures 3 and 4, Figure 6 also makes use of hexagonal binning [32] in the scatter plots, with darker colors indicating greater density of points.) At any given weight parameter value (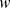 in Equation 18), there is no direct relationship between JED and correlation, so JED cannot be used as a direct proxy for correlation. However, looking across a range of weight parameter values, a general relationship can be observed, allowing general statements about the dependence level induced by a given preprocessing method. In this context, we think of MINDEP for different weight values 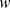 as being different preprocessing methods. Using weight 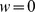 in MINDEP, there is no dependence introduced, and both JED and correlation (between expression estimates for a gene on array pairs) are 0. As the weight parameter increases towards 1, the correlations overall increase, with the main distribution of correlation values centering around 0.7 for the higher weights. At the same time, as the weight parameter increases towards 1, the JED values' range increases, with larger JED values becoming more common. In other words, the proliferation of larger JED values is indicative of higher underlying correlations being possible. Such a general relationship can only be shown explicitly for a preprocessing method like MINDEP, where correlation (scaled covariance) can be calculated.

## Discussion

Throughout this paper, we have used the term “numerical dependence” as a convenient descriptive term to distinguish from “statistical dependence.” In reality the JED measure is also related to the notion of robustness (of the gene expression estimate on one array to the inclusion/exclusion of another array for/from consideration). In general, it is not always clear how to statistically define robustness [24], and in the specific case of the JED measure, there is no direct translation to correlation. We investigated several approaches to incorporate our JED measure into an estimate of the covariance matrix 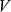 (Equation 15) for this purpose, but finally concluded that while numerical dependence can be assessed via the JED measure, it can not be used to define statistical dependence in a general way. For that reason, we do not present any method to account for numerical dependence in a test for differential expression. We do note, however, that some available tests for differential expression use probe-level rather than fully preprocessed data, so the dependence issue is less of a concern for those methods, which are particularly well-suited to small-sample studies [33].

In presenting the JED here, we are very careful to state that we only propose to use the JED measure as a diagnostic comparison of preprocessing methods, and not for inference; in fact we emphasize that it can not be contorted to fit the purpose of inference. The results of Figure 6 indicate that while JED values cannot be used as a direct proxy for correlation values for any given array pair for any given gene, the JED can be used as a diagnostic to assess the relative amounts of dependence induced by various preprocessing methods.

The JED measure does not estimate a particular parameter – it only provides a summary of the amount of numerical information shared between arrays in calculating gene expression estimates. Because it does not pertain to a defined parameter (but rather to the notion of robustness), the JED measure does not lend itself to hypothesis testing or thresholds of statistical significance. For this reason, we do not propose cut-offs for “acceptable” JED values. Such thresholds (perhaps for “failure” of a preprocessing method) would of necessity be subjective because the relationship between JED and statistical correlation will depend on the preprocessing method (and not, in general, be known for the most common preprocessing methods). Instead, we propose and present here an objective evaluation of several preprocessing methods by demonstrating their JED performance on multiple real data sets (in the Results section). A wider range (and larger extremes) in JED values is indicative of greater induced numerical dependence. A gene's JED value for a pair of arrays is interpreted as the average percent change in the gene's expression value based on the inclusion or exclusion of each array, and as such, is an interesting diagnostic in its own right (even without incorporation to a test of differential expression). For example, there are some moderately large JED values (around 0.35) in Figure 4B for genes with expression values around 10; the interpretation of these values is that those genes (after PLIER preprocessing) are subject to about 35% average change in expression based on the inclusion or exclusion of some arrays. Ideally, there would be no numerical dependence induced by preprocessing (JED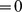). However, it is not only the existence of extreme values that concern us, but the abundance of large JED values (or the skewing of the JED distribution towards 1) in some preprocessing methods (Figures 2 and 6) that we note with alarm.

The JED measure presented here can be used to comparatively assess the numerical independence of gene expression summaries from any given preprocessing method. In fact, this is the primary strength of the JED measure. Better-known measures of dependence or correlation require knowledge of distributions or well-known statistical properties of estimates, which is not the case for most common preprocessing methods. For example, consider 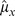 and 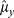 as estimated expression values for a given gene on arrays 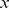 and 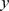, respectively. Just to calculate the simple covariance 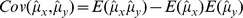 requires knowledge of either the [non-empirical] probability distribution for 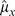, 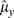, and 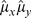, or else the statistical properties of the vector 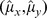. These are known for the contrived MINDEP method presented here. However, for most commonly-used preprocessing methods, the probability distribution of estimated expression values is not known (or even assumed!), and their statistical properties are not well-known. The JED measure provides a way to quickly summarize some notion of dependence between arrays for any preprocessing method, with no need to know its distributional properties.

We emphasize that the JED measure is not a diagnostic of arrays or samples or genes, but of preprocessing methods. We do not propose (and in fact actively discourage) the use of JED for other purposes such as, for example, to identify significantly correlated arrays. While it could be shown for some preprocessing methods that lower JED values roughly correspond to higher correlations between arrays, we discourage this approach (and do not show the results of a simulation we considered to address this very point) for two reasons. First, if an analysis objective is to identify significantly correlated arrays, it is conceptually and computationally far more simple to look at scatterplots of log-scale PM (between pairs of arrays) or something similar than to use the JED. Second, the JED measure has no basis for inference; it is simply a descriptive statistic that, viewed across many genes in several microarray studies (as we have done here), provides insight to the relative levels of numerical dependence induced by various preprocessing methods. This is its sole intended purpose. The JED's performance (in assessing relative amounts of numerical dependence from various preprocessing methods) can only be assessed by repeated application to several data sets, as we have done here. Any JED-based inference would, of necessity, require knowledge of the statistical properties of the JED measure. As discussed in the “JED and Correlation” section above as well as the preceding paragraph, such knowledge is unavailable for the commonly-used preprocessing methods, but fortunately such knowledge is also unnecessary for using the JED in its intended purpose.

Even though a preprocessing method may demonstrate stricter independence in the JED sense (such as MAS5 in Figure 2), it is not necessarily the “best” preprocessing method. Other measures such as bias and performance on spike-in datasets [34], [35] are important to consider in the selection of a preprocessing method. We do not recommend any particular method here, but note in passing that the popular RMA method demonstrates only modest numerical dependence in comparison to some other methods currently used in the literature (Figure 2).

Newer technologies such as RNA-Seq are of course becoming more common for gene expression experiments, and statistical methods are being developed for their appropriate analysis [36], [37]. However, microarrays remain a vital research tool in many fields where an organism's transcriptome is fully defined, and funds are limited. Furthermore, the vast archives of publicly-available microarray data (most notably, NCBI's GEO [38]) serve as a rich resource for targeted hypothesis generation and validation in modern studies, and their use is active and ongoing [39]. The appropriate analysis of microarray data (including appropriate application of independence assumptions) will continue to lead to new biological insights.

Motivated by a desire to avoid lost statistical power (as demonstrated by Figure 1 and the Introduction section above) in tests for differential expression, we encourage the use of preprocessing methods with lower numerical dependence. The JED measure here can assess some notion of dependence for any preprocessing method, even when the distributional properties of the method's expression values are unknown. By doing so, we wish to draw attention to the underlying assumption of (between-array) independence in gene expression summaries for tests of differential expression. This issue of (between-array) independence has received little if any attention in the literature, and researchers working with gene expression data should not take these properties for granted, or they risk unnecessarily losing statistical power.

## Supporting Information

Text S1 **A .txt file providing the R code to obtain this JED measure** (**with an example**)**.**
(TXT)

## References

[1] Lockhart DJ, Dong H, Byrne MC, Follettie MT, Gallo MV, et al (1996) Expression monitoring by hybridization to high-density oligonucleotide arrays. Nature Biotechnology 14. 10.1038/nbt1296-1675 9634850

[2] Craig BA, Black MA, Doerge RW (2003) Gene expression data: The technology and statistical analysis. Journal of Agricultural, Biological, and Environmental Statistics 8: 1–28.

[3] Bolstad BM (2004) Low-Level Analysis of High-Density Oligonucleotide Array Data: Background, Normalization, and Summarization. Ph.D. thesis, University of California, Berkeley, Department of Statistics.

[4] Bolstad BM, Irizarry RA, Gautier L, Wu Z (2005) Preprocessing high-density oligonucleotide arrays. In: Gentleman R, Carey VJ, Huber W, Irizarry RA, Dudoit S, editors, Bioinformatics and Computational Biology Solutions Using R and Bioconductor, New York: Springer.

[5] Affymetrix, Santa Clara (2001) CA (2001) Affymetrix Microarray Suite User's Guide Version 5.0.

[6] Affymetrix, Santa Clara (2002) CA (2002) Statistical Algorithms Description Document. Accessed 14 February 2012 at www.affymetrix.com/support/technical/whitepapers/sadd whitepaper.pdf..

[7] Li C, Wong WH (2001) Model-based analysis of oligonucleotide arrays: Expression index computation and outlier detection. Proceedings of the National Academy of Science 98: 31–36. 10.1073/pnas.011404098 PMC14539 11134512

[8] Li C, Wong WH (2001) Model-based analysis of oligonucleotide arrays: Model validation, design issues, and standard error application. Genome Biology 2. 10.1186/gb-2001-2-8-research0032 PMC55329 11532216

[9] Li C, Wong WH (2003) DNA-Chip Analyzer (dChip). In: Parmigiani G, Garret ES, Irizarry RA, Zezer SL, editors, The Analysis of Gene Expression Data: Methods and Software, New York: Springer.

[10] Irizarry RA, Bolstad BM, Collin F, Cope LM, Hobbs B, et al (2003) Summaries of Affymetrix GeneChip probe level data. Nucleic Acids Research 31: e14. 12582260 10.1093/nar/gng015 PMC150247

[11] Wu Z, Irizarry RA, Gentleman R, Martinez-Murillo F, Spencer F (2004) A model-based background adjustment for oligonucleotide expression arrays. Journal of the American Statistical Association 99: 909–919.

[12] Affymetrix, Santa Clara (2005) CA (2005) Technical Note: Guide to Probe Log-arithmic Intensity (PLIER) Estimation. Accessed 14 February 2012 at www.affymetrix.com/support/technical/technotes/plier technote.pdf..

[13] Therneau TM, Ballman KV (2008) What does PLIER really do? Cancer Informatics 6: 423–431. 19259420 PMC2623311

[14] Liu X, Milo M, Lawrence ND, Rattray M (2005) A tractable probabilistic model for Affymetrix probe-level analysis across multiple chips. Bioinformatics 21: 3637–3644. 16020470 10.1093/bioinformatics/bti583

[15] Liu X, Milo M, Lawrence ND, Rattray M (2006) Probe-level measurement error improves accuracy in detecting differential gene expression. Bioinformatics 22: 2107–2113. 16820429 10.1093/bioinformatics/btl361

[16] Liu X, Lin KK, Andersen B, Rattray M (2007) Including probe-level uncertainty in model-based gene expression clustering. BMC Bioinformatics 8. 10.1186/1471-2105-8-98 PMC1847531 17376221

[17] Gentleman RC, Carey VJ, Bates BM, Bolstad B, Dettling M, et al (2004) Bioconductor: Open software development for computational biology and bioinformatics. Genome Biology 5: R80. 15461798 10.1186/gb-2004-5-10-r80 PMC545600

[18] R Development Core Team (2011) R: A Language and Environment for Statistical Computing. R Foundation for Statistical Computing, Vienna, Austria. Available: http://www.R-project.org. ISBN 3-900051-07-0..

[19] Qiu X, Klebanov L, Yakovlev A (2005) Correlation between gene expression levels and limitations of the empirical bayes methodology for finding differentially expressed genes. Statistical Applications in Genetics and Molecular Biology 4: 34. 10.2202/1544-6115.1157 16646853

[20] Zhu D, Li Y, Li H (2007) Multivariate correlation estimator for inferring functional relationships from replicated genome-wide data. Bioinformatics 23: 2298–2305. 17586543 10.1093/bioinformatics/btm328

[21] Smyth GK (2004) Linear models and empirical Bayes methods for assessing differential expression in microarray experiments. Statistical Applications in Genetics and Molecular Biology 3: 3. 10.2202/1544-6115.1027 16646809

[22] Tusher VG, Tibshirani R, Chu G (2001) Significance analysis of microarrays applied to the ionizing radiation response. Proceedings of the National Academy of Science 98: 5116–5121. 10.1073/pnas.091062498 PMC33173 11309499

[23] Seber GAF (1977) Linear Regression Analysis. New York: John Wiley and Sons.

[24] Rey WJJ (1983) Introduction to Robust and Quasi-Robust Statistical Methods. Berlin: Springer-Verlag.

[25] Hoy M, Westad F, Martens H (2004) Improved jackknife variance estimates of bilinear model parameters. In: Antoch J, editor, COMPSTAT: Proceedings in Computational Statistics, Heidelberg Germany: Springer. 261–276.

[26] Nicholas G (2007) A method for finding standard error estimates for RMA expression levels using bootstrap. MS Thesis, Utah State University, Department of Mathematics and Statistics.

[27] Affymetrix (2000) Latin square data for expression algorithm assessment. Available: http://www.affymetrix.com/support/technical/sample data/datasets.affx. Accessed 2012 Feb 14..

[28] Zhu Q, Miecznikowski JC, Halfon MS (2010) Preferred analysis methods for Affymetrix GeneChips. II. An expanded, balanced, wholly-defined spike-in dataset. BMC Bioinformatics 11: 285. 20507584 10.1186/1471-2105-11-285 PMC2897828

[29] Choe SE, Boutros M, Michelson AM, Church GM, Halfon MS (2005) Preferred analysis methods for Affymetrix GeneChips revealed by a wholly defined control dataset. Genome Biology 6. (R16).. 10.1186/gb-2005-6-2-r16 PMC551536 15693945

[30] Hevel JM, Olson-Buelow LC, Ganesan B, Stevens JR, Hardman JP, et al (2008) Novel functional view of the crocidolite asbestos-treated a549 human lung epithelial transcriptome reveals an intricate network of pathways with opposing functions. BMC Genomics 9: 376. 18687144 10.1186/1471-2164-9-376 PMC2533023

[31] Tukey J (1977) Exploratory Data Analysis. Reading, MA: Addison-Wesley.

[32] Carr DB, Littlefield RJ, Nicholson WL, Littlefield JS (1987) Scatterplot matrix techniques for large n. Journal of the American Statistical Association 83: 424–436.

[33] Stevens JR, Bell JL, Aston KI, White KL (2009) A comparison of probe-level and probeset models for small-sample gene expression data. BMC Bioinformatics 11: 281. 10.1186/1471-2105-11-281 PMC2901368 20504334

[34] Cope LM, Irizarry RA, Jaffee HA, Wu Z, Speed TP (2004) A benchmark for Affymetrix GeneChip expression measures. Bioinformatics 20: 323–331. 14960458 10.1093/bioinformatics/btg410

[35] Irizarry RA, Wu Z, Jaffee HA (2006) Comparison of Affymetrix GeneChip expression measures. Bioinformatics 22: 789–794. 16410320 10.1093/bioinformatics/btk046

[36] Auer PL, Doerge RW (2010) Statistical design and analysis of RNA sequencing data. Genetics 185: 405–416. 20439781 10.1534/genetics.110.114983 PMC2881125

[37] Auer PL, Doerge RW (2011) A two-stage Poisson model for testing RNA-Seq data. Statistical Applications in Genetics and Molecular Biology 10: 26.

[38] Edgar R, Domrachev M, Lash AE (2002) Gene Expression Omnibus: NCBI gene expression and hybridization array data repository. Nucleic Acids Research 30: 207–210. 11752295 10.1093/nar/30.1.207 PMC99122

[39] Barrett T, Troup DB, Wilhite SE, Ledoux P, Evangelista C, et al (2011) NCBI GEO: archive for functional genomics data sets – 10 years on. Nucleic Acids Research 39(Database issue). D1005–10. 10.1093/nar/gkq1184 PMC3013736 21097893

